# Feasibility of transesophageal phrenic nerve stimulation

**DOI:** 10.1186/s12938-023-01071-5

**Published:** 2023-01-30

**Authors:** Elisa M. Kaufmann, Sven Krause, Lukas Geisshuesler, Olivier Scheidegger, Andreas Haeberlin, Thomas Niederhauser

**Affiliations:** 1 Institute of Human Centered Engineering HuCE, Bern University of Applied Sciences, Biel, Switzerland; 2 Sitem Center for Translational Medicine and Biomedical Entrepreneurship, University of Bern, Bern, Switzerland; 3 Department of Cardiology, Bern University Hospital, University of Bern, Bern, Switzerland; 4 Department Neurology, Bern University Hospital, University of Bern, Bern, Switzerland

**Keywords:** Phrenic nerve stimulation, Diaphragm activation, Critical care, Esophageal catheter, Intensive care unit, Lung and diaphragm protective, Transesophageal stimulation, Ventilation induced diaphragmatic dysfunction, Hospital mortality

## Abstract

**Background:**

Every year, more than 2.5 million critically ill patients in the ICU are dependent on mechanical ventilation. The positive pressure in the lungs generated by the ventilator keeps the diaphragm passive, which can lead to a loss of myofibers within a short time. To prevent ventilator-induced diaphragmatic dysfunction (VIDD), phrenic nerve stimulation may be used.

**Objective:**

The goal of this study is to show the feasibility of transesophageal phrenic nerve stimulation (TEPNS). We hypothesize that selective phrenic nerve stimulation can efficiently activate the diaphragm with reduced co-stimulations.

**Methods:**

An in vitro study in saline solution combined with anatomical findings was performed to investigate relevant stimulation parameters such as inter-electrode spacing, range to target site, or omnidirectional vs. sectioned electrodes. Subsequently, dedicated esophageal electrodes were inserted into a pig and single stimulation pulses were delivered simultaneously with mechanical ventilation. Various stimulation sites and response parameters such as transdiaphragmatic pressure or airway flow were analyzed to establish an appropriate stimulation setting.

**Results:**

Phrenic nerve stimulation with esophageal electrodes has been demonstrated. With a current amplitude of 40 mA, similar response figures of the diaphragm activation as compared to conventional stimulation with needle electrodes at 10mA were observed. Directed electrodes best aligned with the phrenic nerve resulted in up to 16.9 % higher amplitude at the target site in vitro and up to 6 cmH20 higher transdiaphragmatic pressure in vivo as compared to omnidirectional electrodes. The activation efficiency was more sensitive to the stimulation level inside the esophagus than to the inter-electrode spacing. Most effective and selective stimulation was achieved at the level of rib 1 using sectioned electrodes 40 mm apart.

**Conclusion:**

Directed transesophageal phrenic nerve stimulation with single stimuli enabled diaphragm activation. In the future, this method might keep the diaphragm active during, and even support, artificial ventilation. Meanwhile, dedicated sectioned electrodes could be integrated into gastric feeding tubes.

## Introduction

Every year, more than 2.5 million patients in intensive care units (ICU) require mechanical ventilation (MV). These critically ill patients occupy $21\%$ to $39\%$ of ICU units [1]. Although lifesaving, prolonged MV is associated with adverse effects, as the source of ventilation exerts a positive pressure on the lungs while, the diaphragm remains inactive. Due to the lack of diaphragmatic activity, the diaphragm recedes. Six hours of MV in humans already decreases diaphragmatic contractility. After only 18–69 h of MV [2], more than half of the cross-sectional area of the diaphragmatic myofibers is reduced [3], leading to ventilator induced diaphragmatic dysfunction (VIDD). After MV, the respiratory muscle must be slowly re-accustomed to its own breathing, i.e., weaned off the ventilator. VIDD-related complications such as prolonged weaning or weaning failure occur in almost half of patients ventilated for 24 h or longer and can result in an extended stay in the ICU [4]. Shortening weaning and reducing complications associated with MV is highly desirable [5, 6].

Electrical stimulation of the intact phrenic nerves inducing a contraction of the inactive diaphragm may provide a solution. The idea of activating the diaphragm with electrical pulses goes back many years. As early as 1872, Duchenne defined phrenic nerve stimulation (PNS) as physiologically the best way to mimic natural breathing [7], followed by the first study in 1873 to cure asphyxia [8]. Long-term diaphragm pacing as a contemporary therapeutic tool for a patient with primary hypoventilation can be traced back to the pioneering work of Glenn [9], who introduced the first respiratory pacemaker to the market in 1995 [10].

Similar to diaphragmatic pacemakers, various PNS concepts have been investigated to prevent VIDD, but they suffer from major complications. Conventional noninvasive transcutaneous stimulation of the phrenic nerve has manifested no relevant co-stimulations in healthy volunteers [11]. The optimal electrode position varies greatly between patients. Depending on the placement of the electrode array, central venous access is blocked, which most ventilated patients in the ICU require. Studies using transcutaneous PNS in critically ill patients are still lacking. Cervical magnetic phrenic stimulation also resulted in bilateral diaphragm activation [12]. The strong magnetic fields induced by the bulky coils, however, may lead to interferences with the vital sign monitoring and currently lacks selectivity in ICU patients [13]. Invasive PNS was recently tested successfully through a transvenous electrode catheter [14–16]. This application is, however, limited to patients with existing subclavian venous cannulation [15, 17]. Other invasive methods include electrical percutaneous stimulation [18, 19] and direct diaphragm pacing using implanted electrodes [20, 21], where the latter is not suitable for temporary applications in the ICU.

We propose a novel method using multiple esophageal electrodes for PNS to overcome the drawbacks of existing diaphragm activation methods. Directed esophageal electrodes may enable plane-selective stimulation of the phrenic nerve. Esophageal electrodes also profit from a self-cleaning, stable electrochemical environment, allowing for long-term instrumentation [22] with low infection potential [23, 24]. A simple insertion criterion [25] may be adopted from long-term transesophageal ECG recording to omit X-ray-based electrode placement in the future. Furthermore, esophageal electrodes are sensitive to diaphragmatic electromyography (EMG) [26] and, thus, may give a direct feedback of successful diaphragm activation. The present study tests the feasibility of transesophageal PNS (TEPNS) for the activation of the diaphragm.

## Methods

The study was structured in two main parts. First, a dedicated in vitro measurement setup was built and an appropriate TEPNS protocol with customized esophageal electrodes was examined. Second, an in vivo study with a sedated porcine model was performed. TEPNS was optimized for and performed at the level rib 1 and C5 as indicated by preceding anatomical studies and the in vitro experiments. The in vivo trial was approved by the Veterinary Department of the Canton of Bern, Switzerland, and was performed in compliance with the Guide for the Care and Use of Laboratory Animals.

### Multielectrode esophageal catheter

**Fig. 1:**
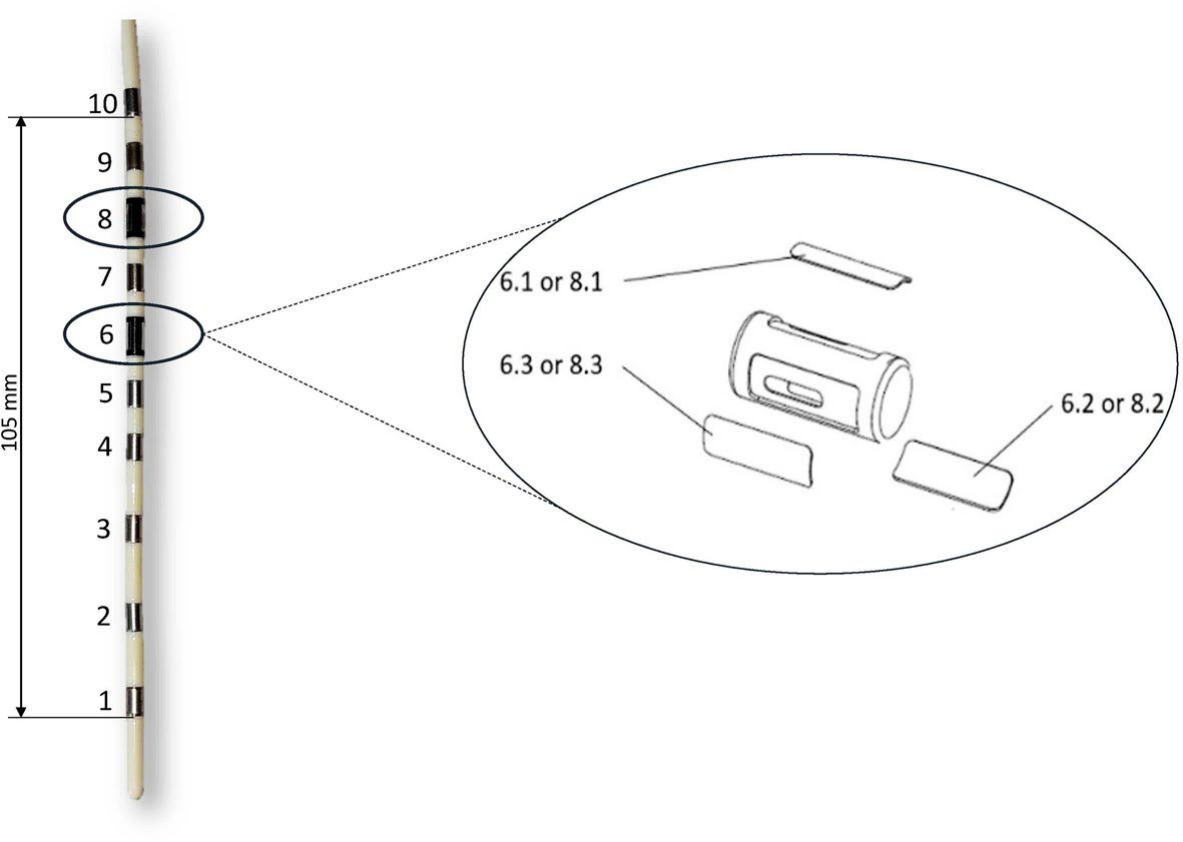
Multipolar esophageal catheter equipped with ten electrode sections. All sections contain omnidirectional electrodes, except sections 6 and 8, which include three directed electrodes in the circumference

A customized multielectrode esophageal catheter was developed with a total of 10 electrode sections that are built partially as round, i.e., omnidirectional electrodes (5 mm length, 3 mm diameter, $47.12\,{\text{mm}}^{2}$ surface, *AISI*304/1.4301) and partially as three sectioned (sections 6 and 8), i.e., directed electrodes in the circumference (5 mm length, 1.83 mm width, $9.15\,{\text{mm}}^{2}$ surface, $AISI 316\,L / 1.4404$) as depicted in Fig. 1. The distance between electrodes 1 to 3 measured 15 mm, the distance between electrodes 4 to 10 was 10 mm each. The thermoplastic polyurethane catheter tube had a total length of 150 mm.

### In vitro setup

**Fig. 2:**
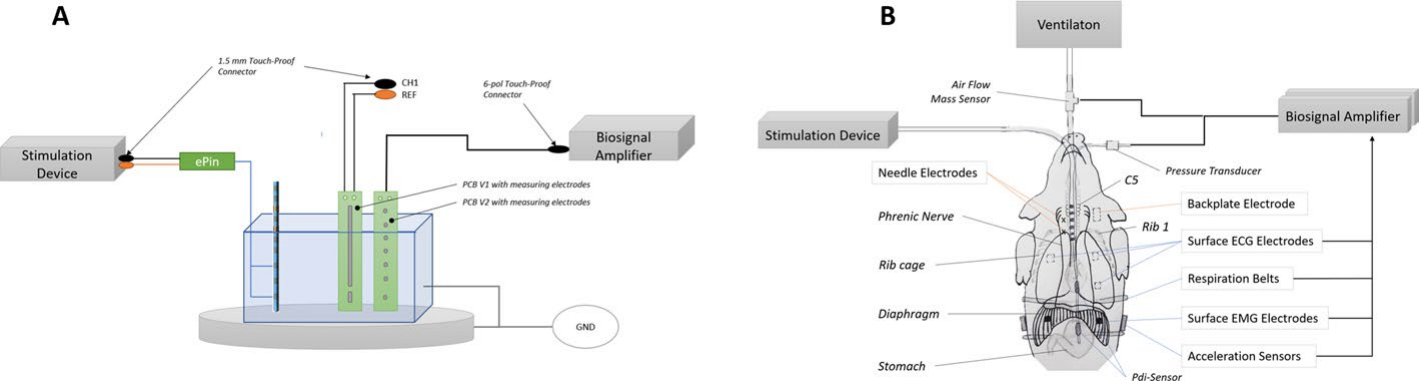
In vitro test setup **A** used for the analysis of stimulation intensities with directed as compared to omnidirectional electrodes and animal trial setup **B** with dedicated stimulation and measurement equipment

In vitro measurements with omnidirectional and directed electrodes were performed. In an isotonic saline solution (0.90$\%$ w/v of NaCl, 308 mOsm/L), the esophageal electrode catheter was placed and aligned with sensing electrodes mimicking the phrenic nerve, as illustrated in Fig. 2A. Bipolar stimulation pulses were generated by the current stimulator DS8R (Digitimer, UK) and delivered from two selected electrodes of the esophageal catheter. The attenuated pulses were measured and digitized by the high-resolution biosignal amplifier g.USBamp (G.TEC, AT). Each measurement was performed 3–5 times with a cathodic square pulse length of 10 mA amplitude and 400$\mu s$ duration. Three stimulation parameters were examined to study their effect on the pulse amplitudes (Appendix 1). The distance between the stimulation and measurement electrodes was varied from 20 mm to 50 mm, in accordance with pig anatomy. The spacing between the stimulation electrodes was changed from 10 mm to 105 mm; meanwhile, omnidirectional and directed electrodes were compared to each other with fixed spacing.

### In vivo trial

An animal trial was done using a 60 kg domestic pig, which was sedated, mechanically ventilated and placed on the back. Anesthesia was administered with Sevoflurane. The multielectrode catheter (Fig. 1) was inserted into the esophagus to the desired positions using real-time X-ray imaging and connected to the stimulation device (Fig. 2). A preliminary anatomical study with pigs [27] showed similar courses of the left and right phrenic nerve and similar branches and connections to the truncus as compared to the human body and was decisive for the choice of the two stimulation positions. For stimulation position 1, the catheter electrode #5 was aligned with the level of rib 1, position 2 was at the level of the nerve origin at vertebrae C6 (electrode #6). In addition, needle electrodes ($0.34\,{\text{mm}}^{2}$ worksurface) were used to puncture the phrenic nerve parasternal to obtain a positive control of PNS and concurrent diaphragmatic activation. The three stimulation sites were characterized and compared based on pressure measurements acquired with a differential pressure transducer (Harvard Apparatus, US) connected to a two-site balloon catheter (Nutrivent, IT), two respiration effort sensors on stretching belts (G.TEC, AT) placed on the thorax and abdomen, respectively, an airflow sensor (Sensirion, CH) and two tri-axial-accelerometer (BIOPAC, US) placed on the left and right side, as shown in Fig. 2B. Single stimulation pulses were delivered immediately after the exhale phase of the mechanical ventilation. The amplitude and duration of stimulation pulses ranged from 10 mA and 200 μs (equals 2000 nC/pulse) to 60 mA and 400 μs (equals 24000 nC/pulse), respectively. MATLAB R2020b (Mathworks, USA) was used for the signal analysis. For all measurement series, median values from five time samples of the measurement device were calculated, focusing on the transdiaphragmatic pressure (Pdi) and the behavior of the left and the right accelerator and the lower respiratory belt.

## Results

### Optimal measurement protocol

**Fig. 3:**
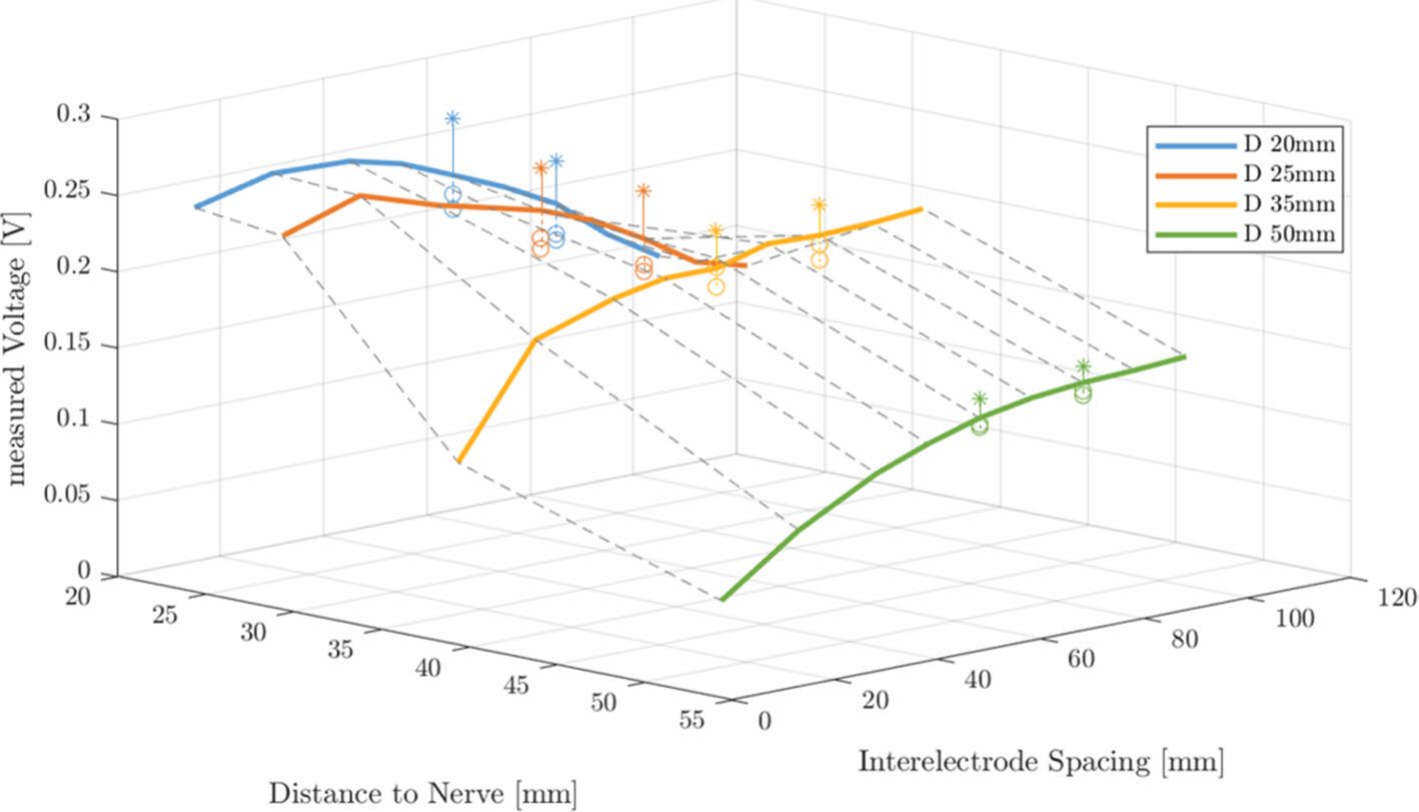
Peak-to-peak amplitude of the measured stimulation pulses at 10mA as a function of the interelectrode spacing, emulated distance to nerve and electrode type. The markers indicate measurements with directed electrodes facing toward the nerve (*) and pointing 120$^\circ$away (o). At the distance of 20–30 mm to the nerve, a smaller interelectrode spacing of 20 mm to 40 mm produces a higher potential as compared to higher spacing. An additional gain of up to 16.9% is measured through aligned electrode segments.

Target intensity of the attenuated stimulation pulses as a function of the interelectrode spacing and distance to the target structure is depicted in Fig. 3. As expected, the measured potential follows the function $f(x) = \dfrac{1}{a(x-b)^{2}+c}$, with *x* being the spacing, $\dfrac{1}{c}$ the maximal intensity at spacing *b,* and *a* the width of the intensity curve keeping the distance constant. A higher distance to the target site incrementally flattens the intensity curve $(\downarrow a)$, decreases the maximal intensity $(\uparrow c)$ that occurs at a higher spacing $(\uparrow b)$. The directed electrodes (current density: $1.092 mA/{\text{mm}}^{2}$) facing the stimulation target achieved 16.9% higher intensity as compared to the omnidirectional electrodes ($0.212 mA/{\text{mm}}^{2}$) for a distance of 20 mm to the target and independent of the spacing. The gain decreases for higher distances to the target and was 9.89% at 50 mm distance. In contrast, directed electrodes not aligned with the measurement electrodes generate up to 10.14% and 4.6% less intensity than omnidirectional electrodes at 20 and 50 mm distance to the target, respectively. If the expected distance of 20–30 mm to the phrenic nerve is considered, the suitable interelectrode spacing for the in vivo trial would be between 20 and 40 mm.

### Esophageal electrode placement

**Fig. 4:**
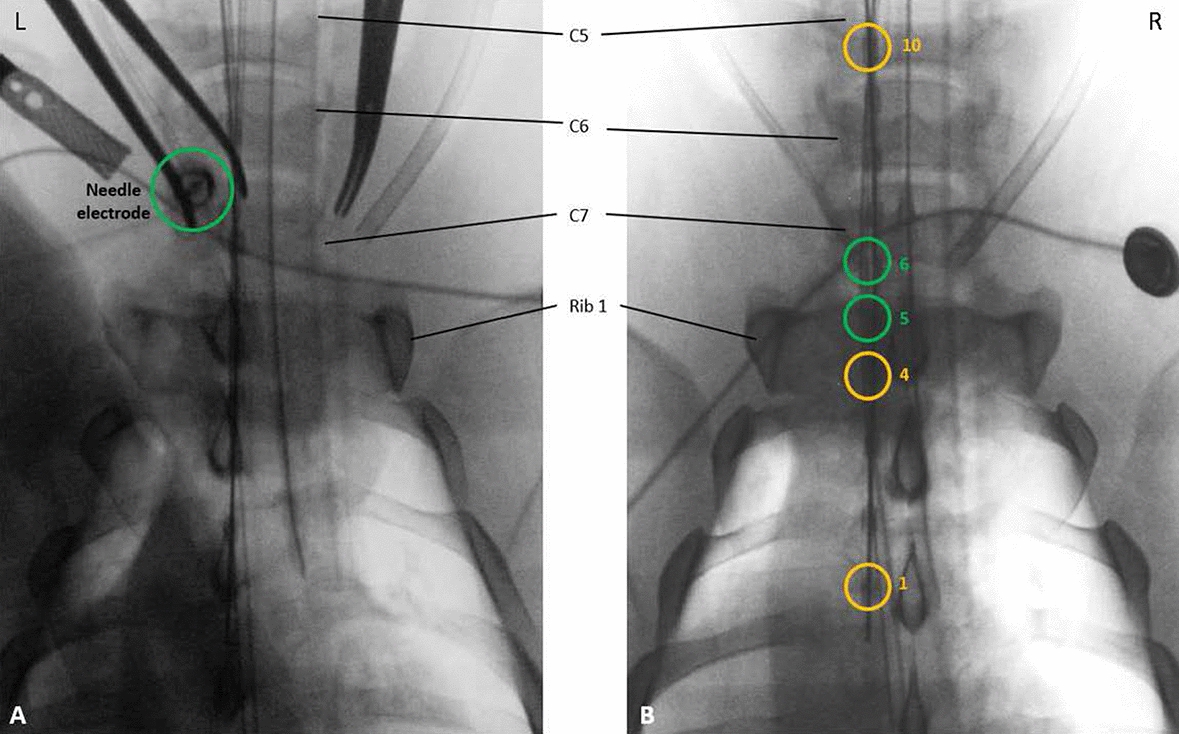
Posterior–anterior X-ray of the pig chest and neck during the trial. The locations of the stimulation electrodes are marked in green. A) Parasternal insertion site of the needle electrode used to puncture the left phrenic nerve at level rib 1 / C7. B) Esophageal electrodes #5 and #6 with the shortest expected distance to the phrenic nerve aligned at the same level, i.e., rib 1 / C7

Figure 4 illustrates the experimental in vivo setting. For the X-ray of the pig’s chest and neck, the pig is lying on its back. The inserted needle electrode is shown in Fig. 4A. Figure 4B shows the position of the esophageal electrodes, with electrode #6 placed at the level of rib 1. This is considered the first zero-position. For the second zero-position, the catheter was moved proximally until electrode #6 was at the level of vertebra C6.

### PNS samples

**Fig. 5:**
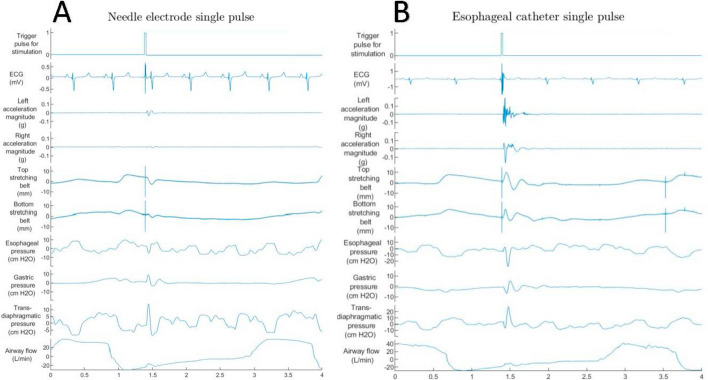
Capture of the multimodal signals after the delivery of a single stimulus with the punctured needle electrode (left) and esophageal electrodes symmetrical to level rib 1 and 40 mm spacing (right). Due to directed TEPNS, similar Pdi values are achieved compared with needle electrode stimulation, but with significantly higher bilateral acceleration values

Fig. 5A shows representative multimodal measurements during stimulation of the left phrenic nerve with a unipolar needle electrode using a current intensity of 10 mA (equals a current density of $39.41 mA/{\text{mm}}^{2}$). As expected, a much higher peak acceleration was recorded for the left as compared to the right side. With the upper respiration belt, a stronger extension of 10.2 mm can be observed as compared to the lower respiration belt achieving 4.9 mm. The Pdi shows a positive peak of 0.4 cmH2O manifesting as remarkable inward flow of about 7.9 L/min superimposing the ventilation pattern. In Fig. 5B, stimulation was performed with the split esophageal electrode aligned to the left nerve with a current amplitude of 40 mA to achieve a similar airflow pattern. The esophageal stimulation with an increased energy resulted in higher bilateral acceleration peaks as well as stronger belt excursion (top and bottom) as compared to the needle stimulation. The induced peak negative pressure in the esophagus of more than 20 cmH2O manifests as Pdi pressure of 12.4 cmH2O and an inward flow of 7.7 L/min simultaneous to the outflow pattern of the ventilation.

**Fig. 6:**
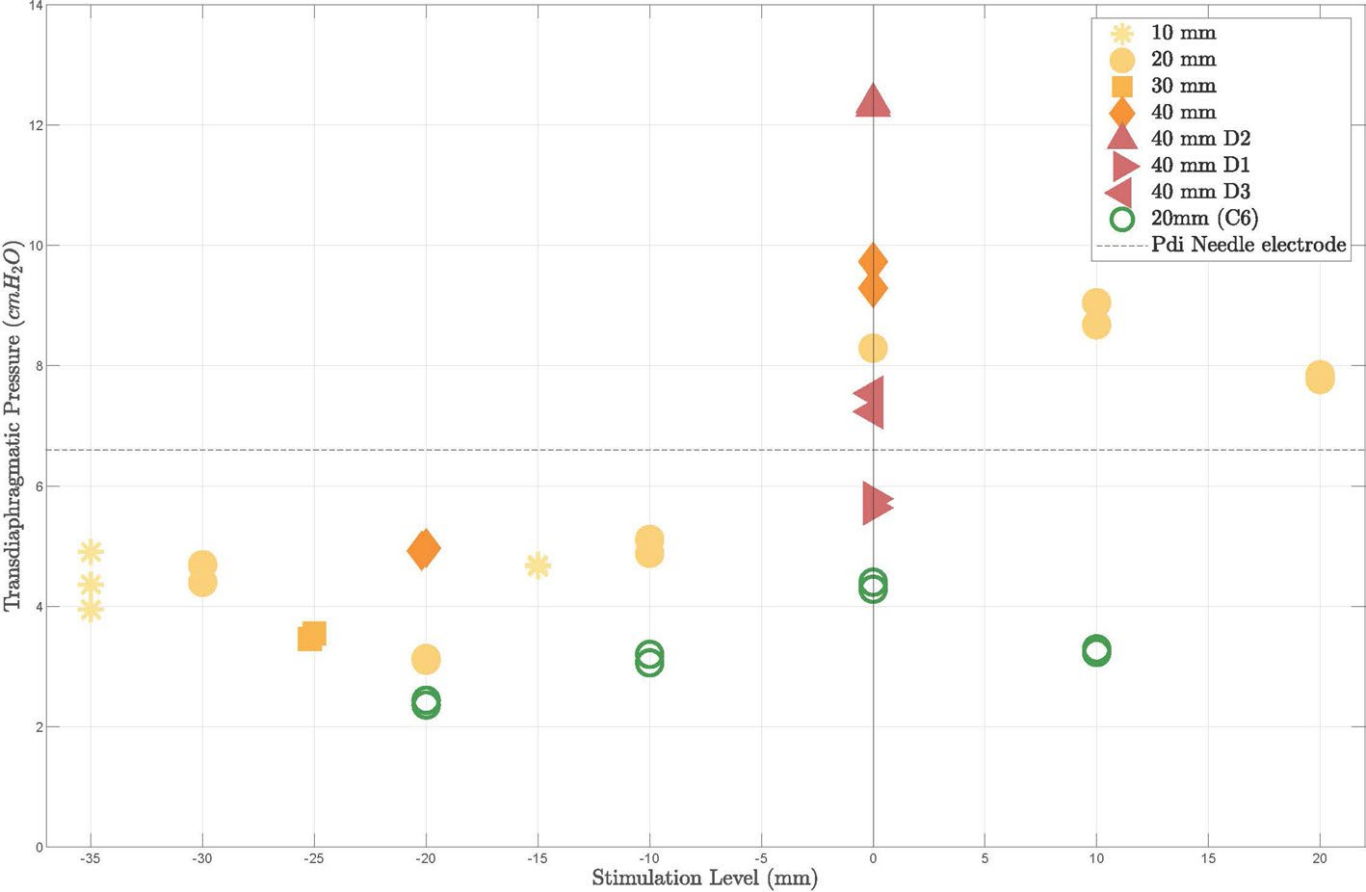
Stimulation-induced Pdi as a function of the esophageal stimulation level around rib 1 (orange shades) and C6 (green), and the electrode spacing (10–40 mm) with a stimulation intensity of 40 mA. The horizontal dashed line indicates phrenic nerve capture with the punctured needle electrode placed at rib 1. The zero level, aligned accordingly to rib 1 or C6, is depicted by a vertical line. A significantly higher Pdi is generated at the stimulation position of rib 1 than at the level of C6. Furthermore, a gain in Pdi can be observed by the best aligned directed electrode as compared to the omnidirectional electrode

**Fig. 7:**
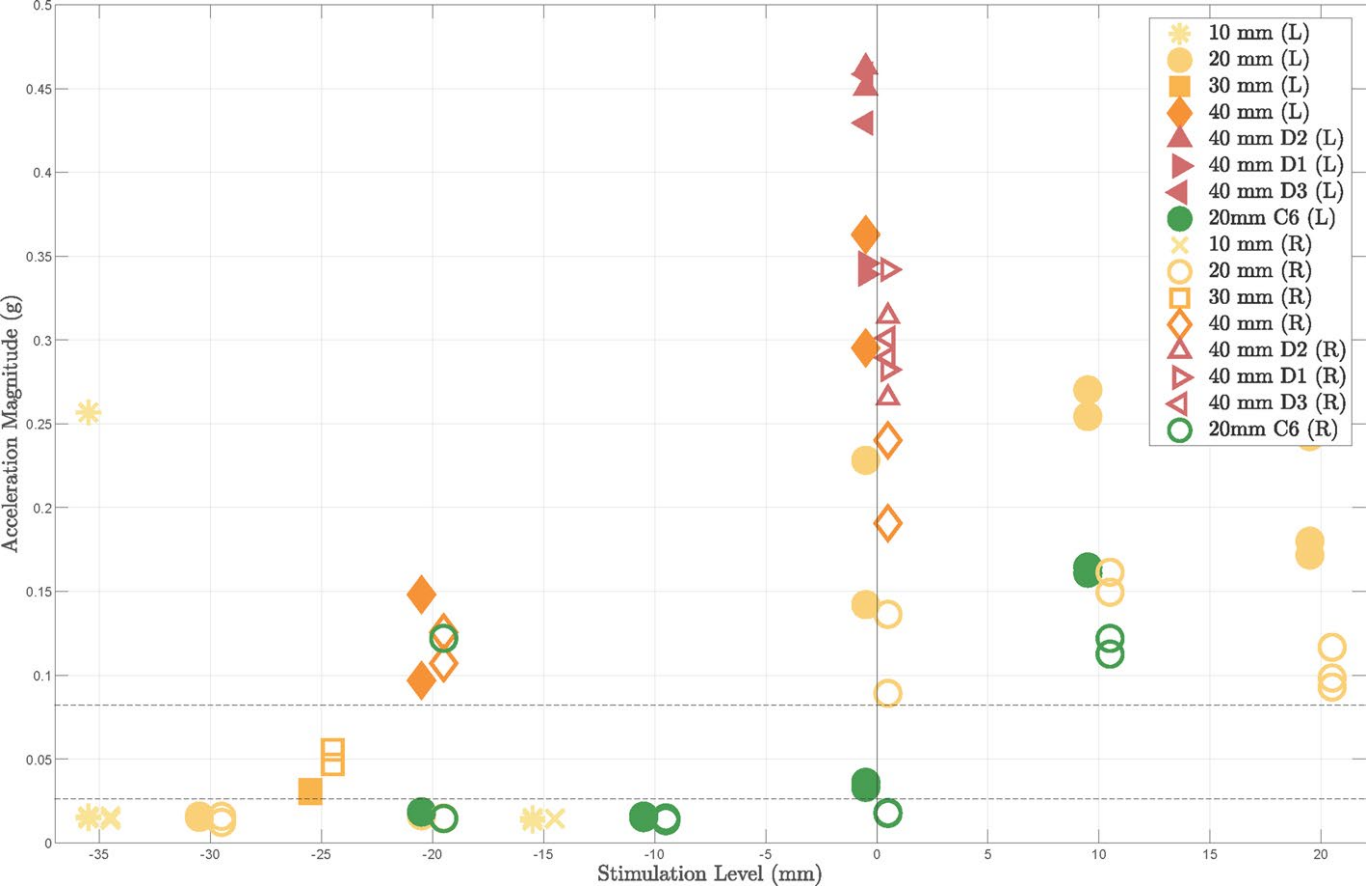
Left (L, filled markers) and right (R, empty markers) acceleration amplitude as a function of the esophageal stimulation level around rip 1 (orange-shades) and C6 (green) as well as the electrode spacing (10–40 mm) with a stimulation intensity of 40 mA. The average left and right acceleration amplitude achieved during stimulation with the needle electrode is indicated as an upper and lower dashed line, respectively. Compared to impulses from the needle electrode, significantly higher acceleration values are measured through stimulation with esophageal electrodes at the level of rib 1, which are as attributed to a higher amount of co-stimulation

### Diaphragm activation and co-stimulation

The stimulation-induced Pdi as a function of the electrode spacing and level in the esophagus is shown in Fig. 6. Impulses delivered with electrode positions more proximal to the reference levels, rib 1 and C6, are given in negative range. Impulses delivered distal to the reference levels, are given in positive range. Similar to the in vitro results (see Fig. 3), a higher Pdi of up to 30% could be induced with the best aligned directed electrode (D2, current density: $4.37 mA/mm^{2}$), as compared to omnidirectional electrodes ($0.85 mA/mm^{2}$) resulting in an average Pdi of 9.5 cmH2O at a spacing of 40 mm. The poorly aligned electrodes induced an even smaller Pdi of 7.5 (D3) cmH2O and 5.8 (D1) cmH2O (max. $-$38.95% with respect to 9.5 cmH2O). With a shorter spacing of 20 mm the highest Pdi pressure of 9.1 cmH2O was achieved by stimulating 10 mm more distal. Overall, symmetrical stimulation around rib 1 induced a considerably higher Pdi than at level C6, for which a maximum of 4.4 cmH2O was obtained with 20 mm electrode spacing. The tidal volume by the MV was 9.72 ml/kg body weight on average. Delivery of single stimuli using directed TEPNS resulted in a tidal volume of up to 0.53 ml/kg, which is comparable to 0.59 ml/kg produced by the needle electrode stimulation (Fig. 5).

The stimulation-induced bilateral acceleration as a function of the electrode spacing and level in the esophagus is shown in Fig. 7. All stimulations at the level of rib 1 produced higher acceleration peaks than with the case of stimulation by needle electrode. Maximal left acceleration was 0.08 g for stimulation with the needle electrode and, at a distance of 40 mm, 0.36 g, 0.46 g and 0.34 g and 0.43 g for stimulation with the omnidirectional, the best aligned directed electrode (D2) and the nonaligned electrodes (D1 and D3), respectively. When dividing the left (filled markers) and right accelerometer values (empty markers), a ratio of $3.11 \pm 0.15$ for stimulation with the needle electrode and, at a distance of 40 mm, a ratio of $1.40 \pm 0.25$, $1.68 \pm 0.06$ and $1.27 \pm 0.13$ for stimulation with the omnidirectional, the best aligned directed electrode (D2) and the nonaligned electrodes (D1 and D3), respectively. At the stimulation position around C6 (green markers), the acceleration values are much lower compared to stimulation around rib 1 with 20 mm spacing. A smaller left-to-right ratio of $1.944 \pm 0.11$ was observed 10 mm more distal to the zero position than at rib 1.

## Discussion

Lung and diaphragm protective MV is highly desirable. This study shows the feasibility of a novel method using electrical pulses delivered from a dedicated esophageal catheter to activate the diaphragm. The preferred stimulation protocol includes a pair of sectioned electrodes with a spacing of 40 mm that has been placed at the level of rib 1 approximately 30 mm from the phrenic nerves. Directed single stimuli applied with an intensity of 40 mA resulted in a similar diaphragm activation pattern as compared to stimuli delivered from an optimally punctured needle electrode with an intensity of 10 mA and are favorable with respect to the resulting Pdi as compared to omnidirectional stimulation.

### Influence of distance to target structure and interelectrode spacing

This study reveals that TEPNS with dedicated electrodes and catheter placement was possible with four times higher current intensity than stimulation with a parasternal electrode placed close to the left phrenic nerve. The punctured electrode, however, provides a much higher current density and most likely lower impedance as compared to the esophageal electrodes. Symmetrical stimulation near rib 1, in particular from 0 to +20 mm distal to rib 1, was more efficient than around C6, i.e., twice the Pdi (9.1 cmH2O compared with 4.4 cmH2O at 20 mm interelectrode distance) and higher left-sided activation were observed, as shown in Figs. 6 and 7. This is most likely due to two main facts: first, both the distance and the amount of intervening tissue between the esophageal electrodes and the phrenic nerves may be higher at level C6 level than rib 1. Both alter the stimulus intensity at the target site, an effect demonstrated by the in vitro experiments shown in Fig. 3 and shown previously [28]. The likelihood of stimulating nerves other than the phrenic nerve also increases, which would explain the smaller left-right acceleration ratio, i.e., the lower stimulation selectivity at level C6. Second, a higher amount of motor fibers of the phrenic nerves are innervated more distal to the nerve origin, which is consistent with other studies [29]. As reported by Watanabe et al, larger electrodes exhibit higher voltages. However, smaller electrodes result in deeper activation and better selectivity. Nevertheless, small electrode spacings diminished the selectivity, confirming the results of this study that at the same current of 40 mA and concurrent higher current density, directional stimulation at a larger spacing of 40 mm resulted in a higher Pdi (30 %) as compared to omnidirectional electrodes.

TEPNS was observed to have a higher sensitivity to the targeted stimulation position (rib 1 vs. C6) than to the inter-electrode spacing. This finding correlates with previous results obtained by Benson et al. for transesophageal atrial stimulation [30]. Their study attributed a smaller effect to the bipolar stimulation with an interelectrode spacing ranging from 15.22 to 28 mm, as compared to the correct catheter placement on the stimulation efficiency. However, the effect of the spacing on the stimulation efficiency is higher when the electrodes are closer to the target structure, as proven by the in vitro experiment (see Fig. 3) and indicated in the in vivo trial (see Fig. 6). As known from the preliminary anatomy study of the pig, a distance from the esophagus to the nerve of about 30 mm was expected at both stimulation positions, level rib 1 and level C6. The electrode spacing of 40 mm, therefore, increased the Pdi only marginally compared to 20 mm. Smaller spacings theoretically allow more precise focus on the stimulation intensity at the target nerve, known from other applications, such as neuromuscular electrical stimulation [31]. In contrast, with a large spacing of, e.g., 105 mm, a more intense stimuli may be achieved at more remote structures (following the voltage curves in Fig. 3) and may also innervate multiple nodes of the phrenic nerve, but the spatial focus of the stimulation is lost. Co-stimulations are more likely to be expected in such cases, as well. As supported by the bilateral acceleration measurements, Fig. 7, much higher co-stimulations are observed by stimulation with esophageal electrodes particularly at a spacing of 40 mm than with the needle electrode. To balance stimulation efficiency and amount of co-stimulations, a smaller spacing of 20 mm is proposed for TEPNS.

### Omnidirectional vs. directional stimulation

Variations in capture thresholds between differently oriented nerve fibers can be highlighted by sectioned electrodes, which suggests a higher sensitivity to directed stimulation pulses as proposed by other studies [32]. Using finite element models, Grill et al demonstrated that the size of the electrode circumference significantly varied the current density on the electrode surface. Therefore, the stimulation efficiency can be increased by 20% for axons parallel to the electrode and by 35% for axons perpendicular to the electrode. In addition to the spatial selective nerve, the in-vitro measurements (see Fig. 3) demonstrated an increase of 16.9 % in voltage amplitude by the target-directed electrode as compared to the omnidirectional electrodes with the same spacing. In case the stimulation was performed with electrodes facing $120^\circ$ to the target and having a spacing of 85 mm, the measured voltage amplitude was almost the same as when stimulating with a smaller spacing of 45 mm to 55 mm using directed electrodes, i.e., with almost half the spacing. This implies that stimulation with a directed electrode not only increases the current density (charge), i.e., improves the focus of the stimulation energy to the phrenic nerve, but also reduces co-stimulation of the surrounding tissue or other nerves. Although the orientation of the sectioned electrodes could not be determined (see Fig. 4) during the animal trial, the pressure and acceleration data obtained support this hypothesis. The directed electrodes mostly facing one of the phrenic nerves increased the Pdi by a factor 1/3 compared to stimulation with the omnidirectional electrode. With the electrodes not facing the phrenic nerves, 1/5 and 2/5 lower Pdi were obtained. It is worth noting that the increase and decrease in Pdi is considerably higher than the gain and loss in measured voltage amplitude in vitro. In contrast to the saline solution, which manifests a homogeneous media with omnidirectional impedance field, the impedance field in vivo may rather be inhomogeneous with respect to the electrode’s circumference. Increasing distance to the electrode potentially results in additional loss of stimulation energy toward non-target tissue [33].

Given the proximity of the sympathetic nervous system, no sympathetic effect of TEPNS was noted throughout the whole animal trial. However, relevant co-stimulations could be observed. Since the single stimuli invoked a weak twitch of the diaphragm (see Fig. 5A for stimulation with an optimally punctured needle electrode), the bilateral acceleration signals reflected a superposition of both, diaphragm activation and co-stimulation, (Figs. 5, 6 and 7). The latter is mainly attributed to stimulation of the brachial plexus leading to a twitching of the extremities. In each stimulation setting, the esophageal electrodes produced higher acceleration values (left and right) than the needle electrode, which is probably related to the higher distance from the nerve and the deteriorated focus. The ratio from the left to the right accelerometer can be used to assess the stimulation selectivity. The high acceleration ratio of $3.11 \pm 0.15$ produced by the needle electrode demonstrated diaphragm activation mainly on the left side through left phrenic nerve stimulation. For the best aligned electrode D2, the accelerometer ratio was about 0.28-$-$0.42 times higher than for the omnidirectional or the nonaligned electrodes D1 and D3, confirming our hypothesis to achieve higher stimulation selectivity with directed electrodes. The observed acceleration values at the level of C6 and using a spacing of 20 mm are comparable to the values achieved with the needle electrode, which speaks for less co-stimulation. However, the small acceleration ratio confirms the lack of selectivity of omnidirectional TEPNS.

### Strength and limitations

This study should be considered as a preliminary study to prove the feasibility of TEPNS. The stimulation protocol was limited to single stimuli,  and TEPNS efficiency and selectivity for diaphragm activation were assessed by Pdi and bilateral acceleration ratio. The single stimuli did not result in a complete diaphragmatic activation that would trigger an inspiratory and expiratory cycle, but rather in a twitch of the muscle. Optimization of a dedicated stimulation protocol, i.e., pulse frequency, duration, amplitude of pulse trains, with respect to physiological diaphragmatic activation was beyond the scope of the present feasibility study, but should be investigated in future animal trials.

Comprehensive anatomical and in vitro experiments were performed preliminary to the in vivo study. Although the proof of TEPNS and concurrent diaphragm activation is based on one animal trial only, the findings with respect to important stimulation parameters such as inter-electrode spacing and directed electrodes are consistent. Moreover, relevant Pdi could repetitively be induced by directed stimulation around rib 1, similar to the stimulation with an invasive needle electrode. Applying the optimal transesophageal setting as suggested by this study, future in vivo trials should perform trains of stimulation pulses that imitate a natural diaphragm contraction and, thus, induce a physiological breathing cycle. It will also be necessary to apply direct diaphragm EMG measurements, such that the strength of inhalation can be recorded as a function of the stimulation intensity and level of diaphragm activation. In the best case, the catheter-based transesophageal stimulation with dedicated electrodes may be equipped with side-sensitive EMG electrodes to record and control the activation efficiency with a single probe.

In addition, the directed electrodes (three in the circumference), could only be aligned with an accuracy of $\pm 60 \text{deg}$ to one of the phrenic nerves. The accuracy of the alignment may be refined and optimized with a novel LCP design and bonding process [34] using a scaffold structure to increase the number of electrodes in the circumference of the catheter. In the future, efficient and selective transesophageal stimulation might make mechanical ventilation more physiological, as well as provide lung- and diaphragm protection, thus minimizing discomfort or the risk of weaning failure and thereby relieving the ICU. The easy-to-place esophageal electrodes might be an elegant solution to be integrated into already in place gastric feeding tubes and, thus, provide a smart system for improved ICU care.

## Conclusion

Transesophageal stimulation by single stimuli enables activation of the diaphragm. The insertion of the stimulation catheter into the esophagus makes the treatment minimally invasive, which is a major advantage over currently available technologies. To cope with geometric variations between individuals, a correct anatomical placement and individual selection of the inter-electrode spacing are relevant factors for PNS. Directed electrodes aligned with the phrenic nerve improve the stimulation efficiency and selectivity, which are relevant factors for future application of this novel method for intensive care patients who require mechanical ventilation.

## Data Availability

Data can be made available upon request after publication through a collaborative process. Please contact the corresponding author for additional information.

## Appendix 1—Parameter settings

Based on various parameter settings, the intensity of the measured voltage amplitude is assessed. The influencing factors of the respective parameters are described below.

**Stimulation amplitude:** To protect the tissue and prevent possible co-stimulation, the current amplitude should be kept as low as possible. In humans, motor nerves are stimulated percutaneously with about 10–20 mA. Direct muscle stimulation from 60–80 mA, stimulation directly on the nerve with 1–2 mA. Since various muscle layers and connective tissue types have to be overcome from the esophagus, a cathodic square stimulus of 10 mA is used for the initial situation. In addition, an amplitude of 10 mA is suitable, as the available measuring devices do not exceed the measuring limit at close distances to the nerve.

**Distance between the stimulation electrode and the nerve:** To examine the influence of the distance between the stimulation site and the nerve more precisely, the measuring electrodes on the PCB (imitation of the nerve) can be moved. The distances to the stimulation electrode can be varied from 10 mm to 100 mm. These correspond to the distances that will also be found in the in vivo experiment. Due to the anatomy of pigs, special attention should be paid to distances of 20 mm–40 mm. As a starting point, the measurements are carried out with a distance to the nerve of 35 mm.

**Inter-electrode spacing:** The effect of the distance between the electrodes was evaluated for a distance between the electrodes of 10 mm to 105 mm in 10 and 15 mm steps. The electrode configuration was always selected in relation to electrode 1 on the tip of catheter to check the minimum and maximum spacing under the same conditions. In addition, current impulses were delivered by connected split electrodes, where only the electrode segments EL 8.1, 8.2 and 8.3 are connected, as in-plane-stimulation. All these electrode configurations correspond to longitudinal stimulation of the nerves. Only the in-plane-stimulation, leads to transversal nerve stimulation.
